# Upregulation of histamine receptor H1 promotes tumor progression and contributes to poor prognosis in hepatocellular carcinoma

**DOI:** 10.1038/s41388-019-1093-y

**Published:** 2019-11-18

**Authors:** Jing Zhao, Yiran Hou, Chun Yin, Jing Hu, Tian Gao, Xiaojun Huang, Xiaohong Zhang, Jinliang Xing, Jiaze An, Shaogui Wan, Jibin Li

**Affiliations:** 1 Center for Molecular Pathology, First Affiliated Hospital, Gannan Medical University, Ganzhou, Jiangxi 341000 China; 2 State Key Laboratory of Cancer Biology and Experimental Teaching Center of Basic Medicine, Fourth Military Medical University, Xi’an, 710032 China; 3 Medical College of Yan’an University, Yan’an, Shaanxi 716000 China; 4 Department of Gynecology and Obstetrics, Xijing Hospital, Fourth Military Medical University, Xi’an, 710032 China; 5 Department of Hepatobiliary Surgery, Xijing Hospital, Fourth Military Medical University, Xi’an, 710032 China

**Keywords:** Oncogenes, Cell migration, Cancer models, Cell growth, Cell death

## Abstract

H1 histamine receptor (H1HR) belongs to the family of rhodopsin-like G-protein-coupled receptors. Recent studies have shown that H1HR expression is increased in several types of cancer. However, its functional roles in tumor progression remain largely unknown, especially in hepatocellular carcinoma (HCC). We found that H1HR is frequently unregulated in HCC, which is significantly associated with both recurrence-free survival and overall survival in HCC patients. Functional experiments revealed that H1HR promoted both the growth and metastasis of HCC cells by inducing cell cycle progression, formation of lamellipodia, production of matrix metalloproteinase 2, and suppression of cell apoptosis. Activation of cyclic adenosine monophosphate-dependent protein kinase A was found to be involved in H1HR-mediated HCC cell growth and metastasis. In addition, we found that overexpression of H1HR was mainly due to the downregulation of miR-940 in HCC cells. Moreover, the H1HR inhibitor terfenadine significantly suppressed tumor growth and metastasis in an HCC xenograft nude mice model. Our findings demonstrate that H1HR plays a critical role in the growth and metastasis of HCC cells, which provides experimental evidence supporting H1HR as a potential drug target for the treatment of HCC.

## Introduction

Histamine is a ubiquitous messenger molecule with multiple physiological activities that released from neurons, mast cells, and enterochromaffin-like cells [1]. It exerts various biological effects through its four cognate G protein-coupled histamine receptors: H1, H2, H3, and H4 (H1HR, H2HR, H3HR, and H4HR, respectively) [2]. Previous studies have shown that H1 histamine receptor (H1HR) is extensively involved in a wide array of pathological processes of allergy, such as atopic dermatitis, allergic rhinitis, and asthma [3, 4]. Recently, it was observed that H1HR promotes cell proliferation in several cancer types, including astrocytoma and breast cancer [5, 6], suggesting that H1HR is a possible oncoprotein and potential target in cancer therapy. However, the functional roles of H1HR in tumor growth and metastasis remain largely unknown, especially in hepatocellular carcinoma (HCC).

The human H1HR gene encodes an integral membrane protein consisting of 487 amino acids, which predominantly couples to Gαq/11 proteins [7], resulting in the activation of phospholipase C and subsequent release of the secondary response elements inositol trisphosphate (IP3) and diacylglycerol (DAG), followed by the activation of protein kinase C (PKC). In addition, H1HR activation also stimulates adenylyl cyclase (AC) to generate cyclic adenosine monophosphate (cAMP), which in turn activates protein kinase A (PKA) [8]. Activated PKC and PKA phosphorylate a number of substrates involved in cell signal transduction. However, activation of the H1HR signaling pathway is poorly understood in cancer cells.

In this study, we systematically investigated the expression and functional roles of H1HR in HCC as well as its clinical implication. Our study facilitates an understanding of the pathological roles of H1HR and provides experimental evidence for H1HR as a potential therapeutic target in HCC.

## Results

### H1HR is upregulated in HCC tissues and contributes to tumor progression and a poor prognosis

**Fig. 1:**
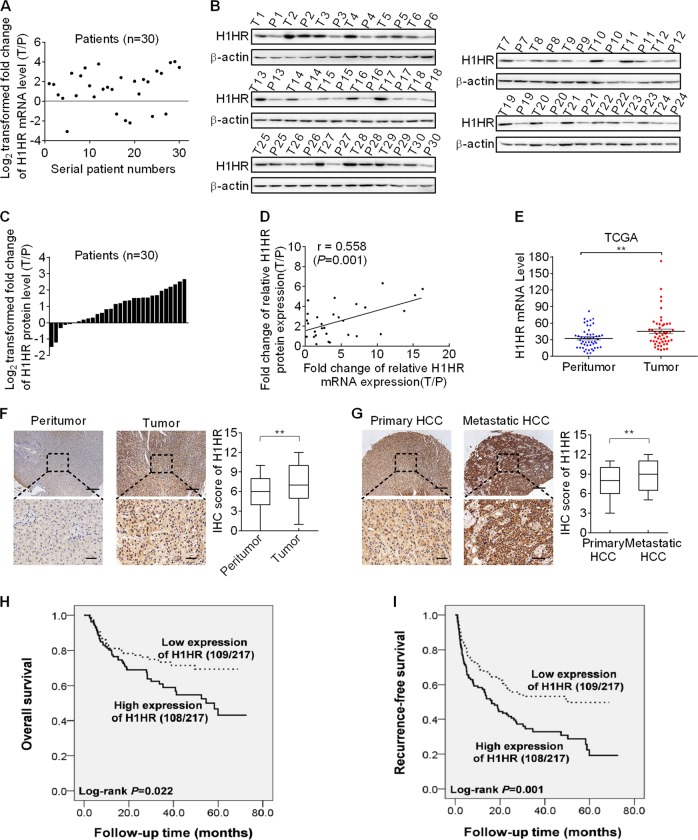
H1HR is upregulated in HCC tissues and associated with tumor progression and poor prognosis. qRT-PCR (**a**) and western blot (**b**) analyses of H1HR expression levels in 30 paired tumor and peritumor tissues from HCC patients (T tumor; P peritumor). **c** The relative expression ratio of tumor to peritumor was log_2_-transformed. **d** Correlation analysis between fold-change of relative mRNA expression (T/P) and protein expression (T/P) of H1HR was conducted in 30 paired tumor and peritumor tissues from HCC patients. Pearson *R*^2^ values are shown. **e** The expression levels of H1HR in HCC tumor and peritumor were statistically analyzed in the TCGA databases. **f** Representative IHC images (left panel) and scores (right panel) of H1HR in 217 paired tumor and peritumor tissues from HCC patients (scale bar, 50 μm). **g** Representative IHC images (left panel) and scores (right panel) of H1HR in 36 unpaired primary HCC and metastatic HCC tissues (scale bar, 50 μm). **h**, **i** Kaplan–Meier survival curves for overall survival (OS) and recurrence-free survival (RFS) stratified by H1HR expression in 217 tumor issues from HCC patients. *P < 0.05; **P < 0.01

We first evaluated the mRNA and protein expression levels of H1HR in 30 paired tumor and peritumor tissues from HCC patients. Both qRT-PCR and western blot analyses showed that H1HR was significantly upregulated in HCC tissues compared with peritumor tissues (Fig. 1a–c). Correlation analysis indicated a significant positive correlation between the mRNA and protein levels for individual patients (Fig. 1d). To further confirm these results, we evaluated the mRNA expression of H1HR using publicly accessible data from The Cancer Genome Atlas (TCGA) database (http://cancergenome.nih.gov). In TCGA cohort of 50 paired HCC tissues, H1HR mRNA expression levels were significantly higher in tumor tissues than in their adjacent normal tissues (Fig. 1e). The expression of H1HR was further evaluated by IHC analysis in another 217-paired tumor and peritumor tissues. As shown in Fig. 1f, the IHC score of H1HR was markedly increased in HCC tissues compared with paired peritumor tissues. In addition, the primary tumor tissues from 36 HCC patients exhibited lower H1HR expression compared with unpaired metastatic tissues from another 36 HCC patients (Fig. 1g). We further evaluated the clinicopathological and prognostic significance of H1HR in HCC patients based on IHC data from 217 tumor tissues. As shown in Supplementary Table 1 H1HR expression was significantly associated with tumor size (*P* < 0.001) and a more advanced tumor–node–metastasis stage (*P* = 0.031). Kaplan–Meier survival analysis showed that HCC patients with high H1HR expression had significantly shorter overall survival (OS) and recurrence-free survival (RFS) than those with low H1HR expression (Fig. 1h, I). Considering that infiltration of myeloid cells has been well-known as a poor prognostic factor in several tumor types [9, 10], multiple staining of H1HR, GPC3 [11–13] (marker of HCC), and CD68 [14, 15] (marker of myeloid cell) by IHC analysis was applied to explore whether H1HR is also expressed in myeloid cells and thus plays a role in the progression of HCC. We found that cells with positive GPC3 and CD68 staining also exhibited H1HR positive staining, indicating that H1HR was expressed in both HCC cells and infiltration myeloid cells (Supplementary Fig. S1A). In addition, bioinformatic analysis based on TCGA database (http://cancergenome.nih.gov) indicated a significant positive correlation between the mRNA expression levels of H1HR and CD68 (*r* = 0.314, *P* = 0.026) (Supplementary Fig. S1B), implying that also myeloid cells present in the tumor microenvironment express H1HR and may contribute to the observed negative impact of H1HR expression on prognosis. Furthermore, TCGA-based survival analysis indicated that overexpressed H1HR predicted a poor survival only in HCC patients with high CD68 expression levels, but not in HCC patients with low CD68 expression levels (Supplementary Fig. S1C, D), which further suggest that increased infiltration of myeloid may aggravate the oncogenic effects exerted by H1HR in HCC. Taken together, these data indicate that H1HR overexpression contributes to the progression of HCC and poor prognosis.

### H1HR promotes HCC cell growth both in vitro and in vivo

**Fig. 2:**
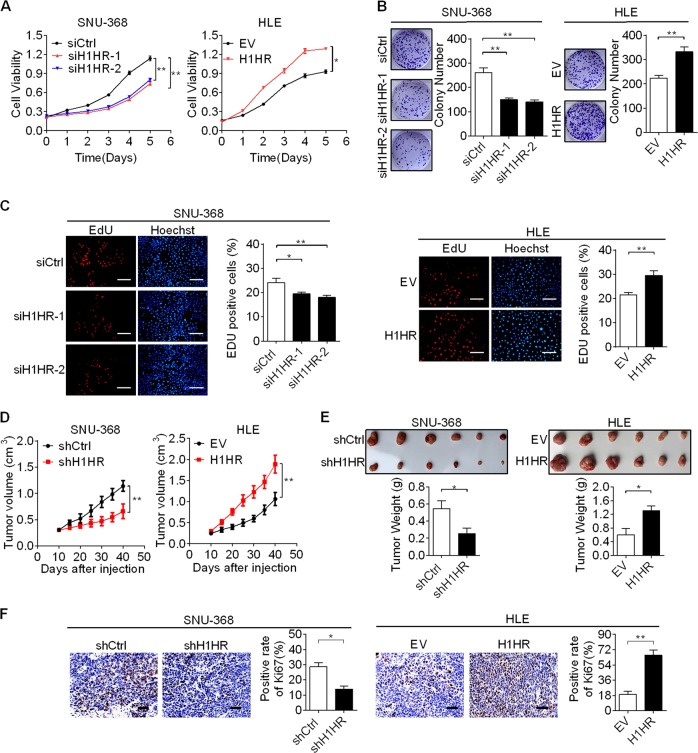
H1HR promotes HCC cell growth both in vitro and in vivo. **a** The MTS cell viability assay was performed in SNU-368 and HLE cells with the indicated treatments (siH1HR-1 and siH1HR-2 siRNAs against H1HR; siCtrl control siRNA; H1HR expression vector encoding H1HR; EV empty vector). **b** Colony formation assay in SNU-368 and HLE cells with treatments as indicated. **c** Cell proliferation ability was evaluated using the EdU incorporation assay in SNU-368 and HLE cells with the indicated treatments (scale bar, 20 μm). **d** The subcutaneous tumor growth curve of HCC cells stably transfected with shH1HR or overexpressing H1HR as indicated in nude mice. **e** Dissected tumors from sacrificed mice (*upper* panel) and their weight (*lower* panel) are shown. **f** Ki-67 staining in tumor tissues of subcutaneous xenografts with the indicated treatments (scale bar, 50 μm). *P < 0.05; **P < 0.01

To assess the potential effects of H1HR on cell growth, a series of in vitro biological experiments were performed with gain-of-function or loss-of-function of H1HR. SNU-368 cells with relatively high H1HR expression and HLE cells with relatively low H1HR expression (Supplementary Fig. S2A, B) were selected to establish cell models with knocked down or overexpression of H1HR (Supplementary Fig. S2C, D). The MTS cell viability assay showed that SNU-368 cells with H1HR knockdown had a much slower growth rate than control cells, whereas HLE cells with H1HR overexpression grew faster than control cells (Fig. 2a). Accordingly, the number of colonies and 5-ethynyl-2′-deoxyuridine (EdU) incorporation were markedly decreased in SNU-368 cells with H1HR knockdown, but were significantly increased in HLE cells with H1HR overexpression compared with controls. (Fig. 2b, c).

To further investigate the in vivo tumourigenic ability of H1HR, we constructed a xenograft nude mice model using SNU-368 and HLE cell lines with stable H1HR knockdown or overexpression (Supplementary Fig. S2E, F). Xenograft tumors developed from SNU-368 cells with stable H1HR knockdown exhibited a significant decrease in growth capacity and less net weight compared with control tumors. In contrast, the growth capacity and net weight of xenografts developed from HLE cells with stable H1HR overexpression were much higher than controls (Fig. 2d, e). Moreover, when compared with controls, those xenografts developed from SNU-368 cells with stable H1HR knockdown exhibited a considerable decrease of positive Ki-67 staining. However, overexpression of H1HR significantly increased Ki-67-positive staining in xenografts developed from HLE cells (Fig. 2f).

### H1HR promotes HCC cell growth by inducing cell cycle progression and suppressing cell apoptosis

**Fig. 3:**
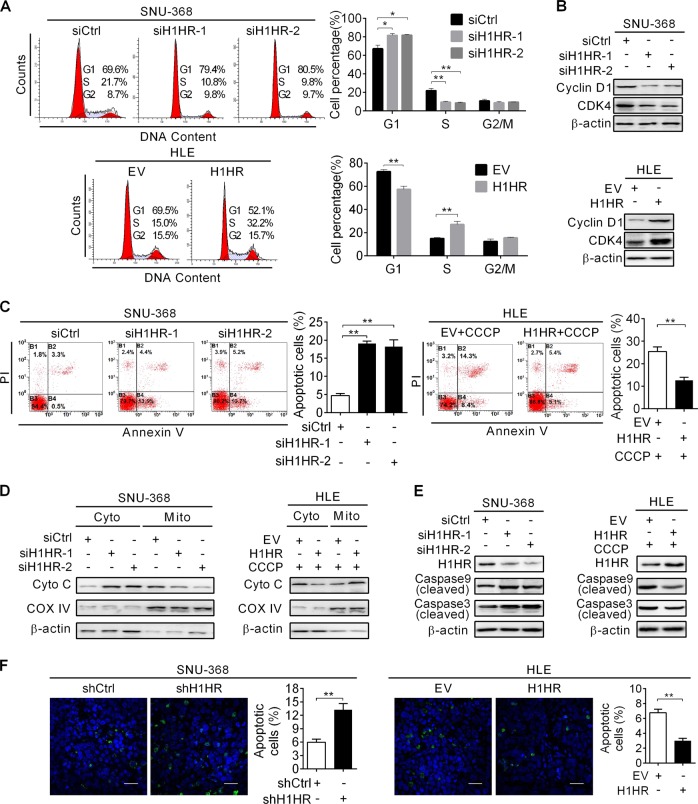
H1HR promotes HCC cell growth by inducing cell cycle progression and reducing cell apoptosis. **a** Cell cycle analysis by flow cytometry in SNU-368 and HLE cells with the indicated treatments. **b** Western blot analysis for cell cycle-related protein expression in SNU-368 and HLE cells with treatment as indicated. **c** Flow cytometry analysis of apoptosis by Annexin V and PI staining in SNU-368 and HLE cells with the indicated treatments. HLE cells were also treated with CCCP (150 μM) for 4 h before apoptosis analysis. **d** Western blot analyses for protein levels of cytochrome c in the cytoplasm and mitochondria of SNU-368 and HLE cells with the indicated treatments. β-actin and cytochrome c oxidase were used as loading controls for the cytoplasm and mitochondria, respectively (Cyto cytoplasm; Mito mitochondria). **e** Western blot analysis of the protein levels of cleaved caspases 9 and 3 in SNU-368 and HLE cells with the indicated treatments. **f** TUNEL staining in tumor tissues of subcutaneous xenografts with the indicated treatments (Blue: Hoechst 33342; Green: TUNEL positive nucleus; scale bar, 20 μm). *P < 0.05; **P < 0.01

To characterize the oncogenic mechanism of H1HR, we further investigated the functional roles of H1HR in cell cycle progression and cell apoptosis. We found that knockdown of H1HR significantly increased the number of SNU-368 cells in the G1 phase, but decreased the number in the S phase compared with the control. Conversely, ectopic expression of H1HR in HLE cells promoted the G1–S cell cycle transition (Fig. 3a). As expected, western blot analysis showed that the protein expression of two major G1–S checkpoint regulators cyclin D1 and cyclin-dependent kinase was decreased in SNU-368 cells with H1HR knockdown, while overexpression of H1HR in HLE cells had opposite effect (Fig. 3b), confirming role of H1HR in promoting cell cycle progression in HCC cells. Next, the effect of H1HR on cell apoptosis was assessed by flow cytometry. We found that knockdown of H1HR significantly enhanced the apoptosis of SNU-368 cells, whereas overexpression of H1HR in HLE cells showed an opposite effect (Fig. 3c). Consistently, cytochrome c release and the cleavage of caspase 9 and 3 were significantly induced by H1HR knockdown, while were significantly inhibited by H1HR overexpression upon CCCP treatment (Fig. 3d, e). We further confirmed the protective role of H1HR against apoptosis in xenograft tumor models. When compared with the control, xenografts developed from SNU-368 cells with stable knockdown of H1HR exhibited a considerable increase in positive TUNEL staining. In contrast, overexpression of H1HR significantly decreased TUNEL-positive staining in xenografts developed from HLE cells (Fig. 3f). Collectively, these data showed that H1HR promoted HCC growth by inducing cell cycle progression and suppressing cell apoptosis.

### H1HR promotes the migration and invasion of HCC cells both in vitro and in vivo

**Fig. 4:**
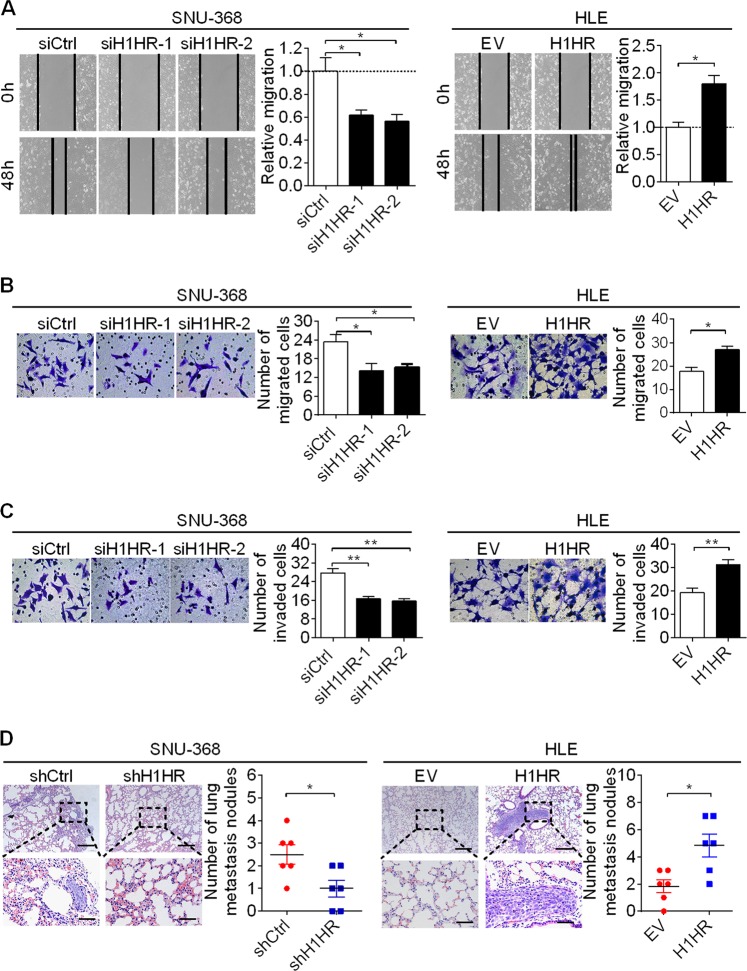
H1HR promotes the migration and invasion of HCC cells both in vitro and in vivo. **a**, **b** Scratch wound healing and transwell migration assays for cell migration abilities in SNU-368 and HLE cells with the indicated treatments. **c** Transwell matrigel invasion assay for cell invasion ability of SNU-368 and HLE cells with the indicated treatments. **d** Incidence of pulmonary metastasis in nude mouse models was demonstrated by hematoxylin and eosin staining (scale bar, 50 μm). *P < 0.05; **P < 0.01

We also investigated the effects of H1HR on the metastasis of HCC cells using both in vitro and in vivo metastasis assays. The scratch wound healing and transwell migration assays showed that knockdown of H1HR significantly inhibited the migratory ability of SNU-368 cells. By contrast, the migratory ability of HLE cells with H1HR overexpression was significantly greater than that of control cells (Fig. 4a, b). The transwell matrigel invasion assay also showed that knockdown of H1HR significantly impaired the invasion of SNU-368 cells, whereas overexpression of in HLE cells had the opposite effect (Fig. 4c). These results indicate that H1HR increases the in vitro migration and invasion abilities of HCC cells. To determine whether H1HR promotes tumor metastasis in vivo, we performed a cancer metastatic assay by injecting SNU-368 and HLE cells with stable H1HR knockdown or overexpression, respectively, into the lateral tail vein of nude mice. We found that mice injected with SNU-368 cells with H1HR knockdown exhibited a dramatic decrease in the incidence of lung metastases, whereas overexpression of H1HR in HLE cells significantly increased the incidence of lung metastases in the metastasis mouse model (Fig. 4d).

### H1HR promotes the migration and invasion of HCC by inducing lamellipodia formation and upregulating matrix metalloproteinase 2

**Fig. 5:**
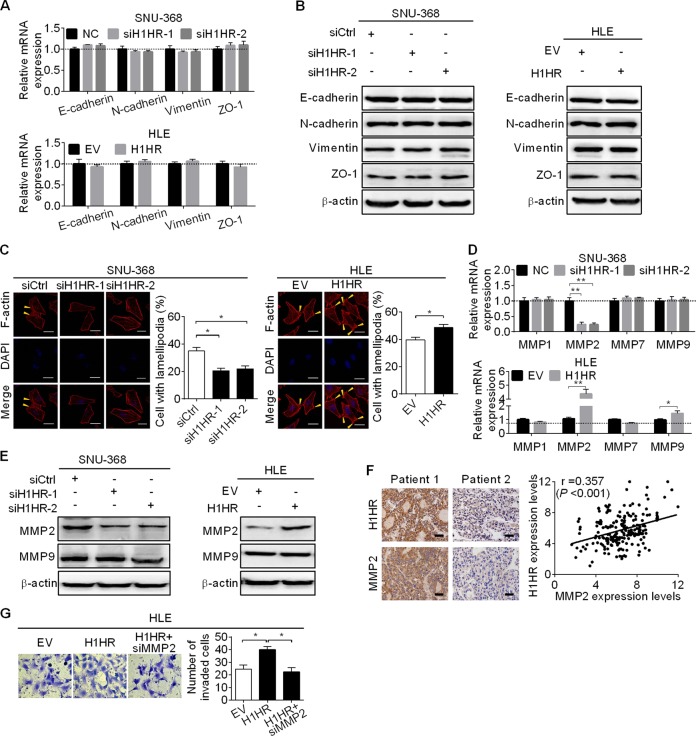
H1HR promotes the migration and invasion of HCC by inducing lamellipodia formation and upregulating MMP-2. **a**, **b** qRT–PCR and western blot analyses for expressions of Epithelial–mesenchymal transition (EMT)-related markers in both SNU-368 and HLE cells with treatment as indicated. **c** Lamellipodia formation was analyzed by F-actin staining in both SNU-368 and HLE cells with the indicated treatments (scale bar, 20 μm). **d** qRT-PCR and western blot analysis for expression levels of MMP-2 in both SNU-368 and HLE cells with the indicated treatments. **f** *Left panel*: representative IHC images of H1HR and MMP2 in 217 HCC tumor tissues (scale bars, 50 μm). *Right panel*: relationship between the expression of H1HR and MMP2. **g** Transwell matrigel invasion assay for cell invasion ability in HLE cells with the indicated treatments (siMMP-2 siRNA against MMP-2; siCtrl control siRNA; H1HR expression vector encoding H1HR; EV empty vector). *P < 0.05; **P < 0.01

The epithelial–mesenchymal transition (EMT) plays a crucial role in tumor metastasis by reducing cell–cell contacts and increasing cell motility. We clarified whether EMT was involved in H1HR-mediated HCC invasion and migration. Quantitative RT-PCR and western blot analysis showed no changes in the expression of epithelial (E-cadherin and zonula occludens-1) or mesenchymal (N-cadherin and vimentin) markers when H1HR was knocked down or overexpressed (Fig. 5a, b), suggesting that H1HR promotes the migration and invasion of HCC not through regulating the EMT. Lamellipodia is a dynamic surface extension of the cell, which plays a pivotal role in cell migration. To explore the mechanisms by which H1HR promotes HCC cell migration, the formation of lamellipodia was examined in SNU-368 and HLE cells with H1HR knockdown or overexpression. F-actin staining revealed that lamellipodia formation was markedly decreased in SNU-368 cells after H1HR was knocked down. In contrast, overexpression of H1HR in HLE cells significantly increased lamellipodia formation (Fig. 5c), suggesting that H1HR may promote the migration of HCC cells by modulating actin rearrangements. To determine how H1HR facilitates the invasion of HCC cells, the expression of four matrix metalloproteinases (MMPs), which are involved in degradation of the basement membrane, were analyzed by qRT-PCR in SNU-368 and HLE cells with H1HR knockdown and overexpression, respectively. Our data showed that only MMP-2 was dramatically downregulated in SNU-368 cells with H1HR knockdown and upregulated in HLE cells with H1HR overexpression (Fig. 5d), which was further confirmed by western blot analysis (Fig. 5e). In addition, we found a significant positive correlation between the IHC scores of H1HR and MMP2 (*r* = 0.357, *P* < 0.001) in 217 HCC tissue samples (Fig. 5f). To determine whether H1HR promotes cell invasion through MMP-2 production, RNA interference was used to knockdown MMP-2 expression in HLE cells with H1HR overexpression (Supplementary Fig. S3). As shown in Fig. 5g, knockdown of MMP-2 was sufficient to inhibit the increase of cell invasion ability induced by H1HR overexpression, suggesting that H1HR may facilitate the invasion of HCC cells by upregulating MMP-2.

### Activation of PKA pathways is involved in H1HR-mediated growth and metastasis of HCC cells

**Fig. 6:**
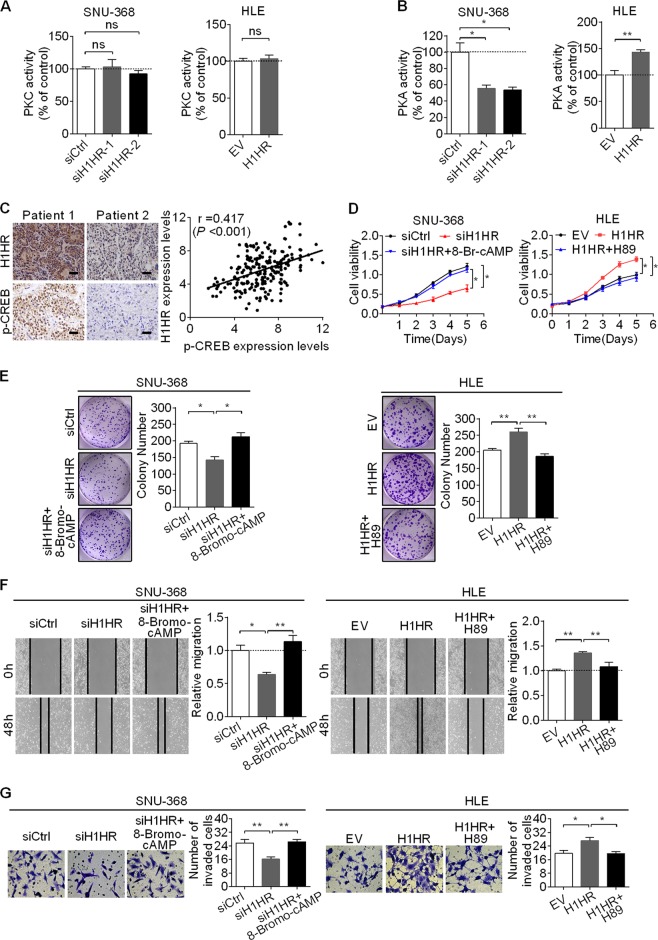
Activation of the PKA pathway is involved in the H1HR-mediated growth and metastasis of HCC. **a**, **b** PKC and PKA kinase activities were analyzed in both SNU-368 and HLE cells with the indicated treatments. **c** *Left panel*: representative IHC images of H1HR and p-CREB in 217 tumor tissues of HCC (scale bars, 50 μm). *Right panel*: relationship between the expression of H1HR and p-CREB was analyzed based on IHC staining. **d**, **e** MTS and colony formation assays for cell growth ability of HCC cells upon treatment with the specific PKA activator 8-bromo-cAMP or inhibitor H89. **f**, **g** Wound healing and transwell matrigel invasion assays for evaluating the migration and invasion abilities of HLE cells upon treatment with H89 or 8-bromo-cAMP. *P < 0.05; **P < 0.01

It is well established that PKC and PKA signaling is commonly triggered by the activation of histamine receptors [16–20]. To test whether PKC and PKA were activated by H1HR in HCC cells, the activities of PKC and PKA were detected in SNU-368 and HLE cells with H1HR knockdown or overexpression. As shown in Fig. 6a, b, the activity of PKA was significantly decreased in SNU-368 cells with H1HR knockdown and significantly increased in HLE cells with H1HR overexpression, while PKC activity remained unchanged, suggesting that the PKA pathway is activated by H1HR in HCC cells. To confirm this observation, we examined the levels of phosphorylated cAMP response element-binding protein (p-CREB, an indicator of PKA activation) in 217 HCC tissue samples, and found a significant positive correlation between the IHC scores of H1HR and p-CREB (*r* = 0.417, *P* < 0.001) (Fig. 6c). Because PKA activation plays a critical role in tumor growth and metastasis, we hypothesized that the oncogenic phenotype induced by H1HR may be associated with activation of the PKA pathway. To test this possibility, we treated HLE cells or SNU-368 cells with the specific PKA inhibitor H89 or PKA activator 8-bromo-cAMP. As shown in Fig. 6d–g, H89 treatment significantly decreased the growth and metastasis of HLE cells induced by H1HR overexpression, whereas 8-bromo-cAMP treatment significantly increased the growth and metastasis of SNU-368 cells suppressed by H1HR knockdown. Collectively, these results indicate that H1HR promotes the growth and metastasis of HCC cells mainly by activating the PKA signaling pathway.

### H1HR overexpression is mainly mediated by downregulation of miR-940

**Fig. 7:**
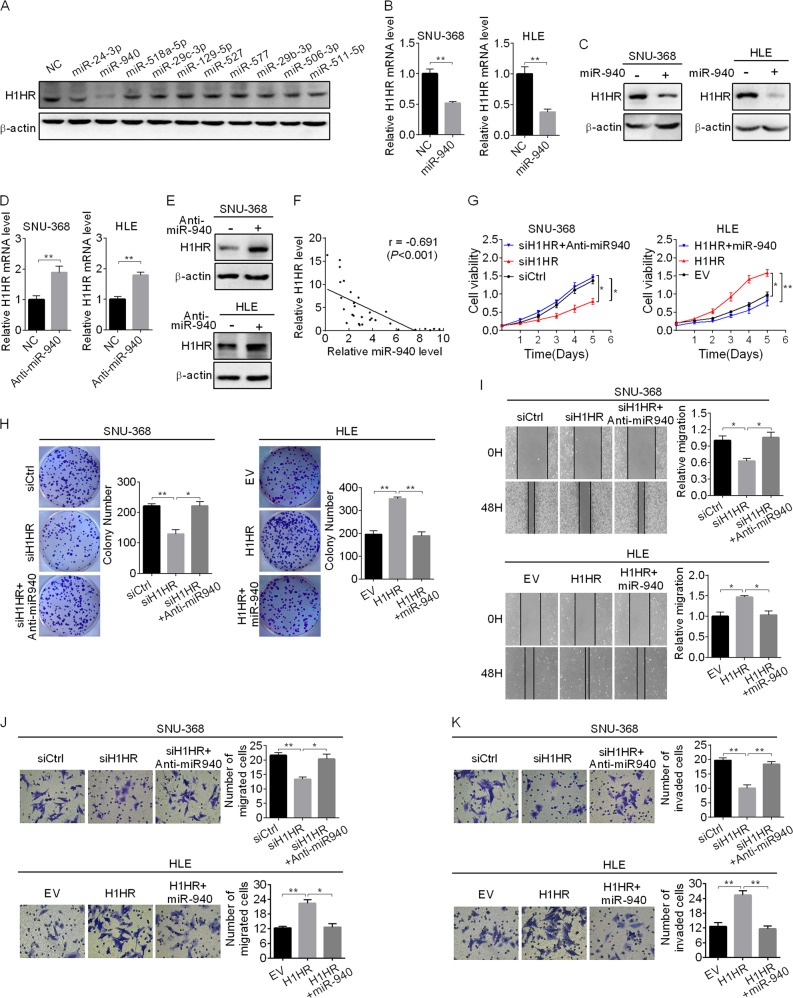
H1HR overexpression is mainly mediated by downregulation of miR-940. **a** Western blot analysis for H1HR expression in SNU-368 transfected with the top ten predicted miRNAs targeting H1HR. **b**, **c** qRT-PCR (left panel) and western blot (right panel) analysis for H1HR expression in SNU-368 and HLE cells transfected with the miR-940 mimics. **d**, **e** qRT-PCR (left panel) and western blot (right panel) analysis for H1HR expression in SNU-368 and HLE cells transfected with the miR-940 inhibitor (Anti-miR940). **f** The correlation between the mRNA levels of H1HR and miR-940 was determined. MTS cell viability (**g**), colony formation assay (**h**), scratch wound healing (**i**), transwell migration (**j**) and transwell matrigel invasion (**k**) assays in SNU-368and HLE cells with treatment as indicated. *P < 0.05

MicroRNA plays an important role in the gene-expression regulation network. To identify potential microRNAs involved in the overexpression of H1HR in HCC, we used microRNA Data Integration Portal-based target prediction programs [21]. Among the top ten predicted miRNAs targeting H1HR (Supplementary Table 2), only miR-940 repressed H1HR expression in SNU-368 cells (Fig. 7a). Real-time PCR and western blot showed that miR-940 significantly downregulated the expression of H1HR in SNU-368 and HLE cells (Fig. 7b, c). In contrast, miR-940 inhibition enhanced H1HR expression in SNU-368 and HLE cells cells (Fig. 7d, e). In addition, a significant negative correlation (*r* = −0.691, *p* < 0.001) between miR-940 and H1HR was found in tumor tissues from 30 HCC patients (Fig. 7f). Moreover, miR-940 mimics greatly attenuated the ability of H1HR to promote the growth and metastasis in HCC cells, whereas miR-940 inhibition increased the growth and metastasis repressed by H1HR knockdown in HCC cells (Fig. 7g–k). These results suggest that miR-940 represses the expression of H1HR and its oncogenic function in HCC cells.

### The H1HR inhibitor terfenadine exhibits therapeutic effects on HCC in vitro and in vivo

**Fig. 8:**
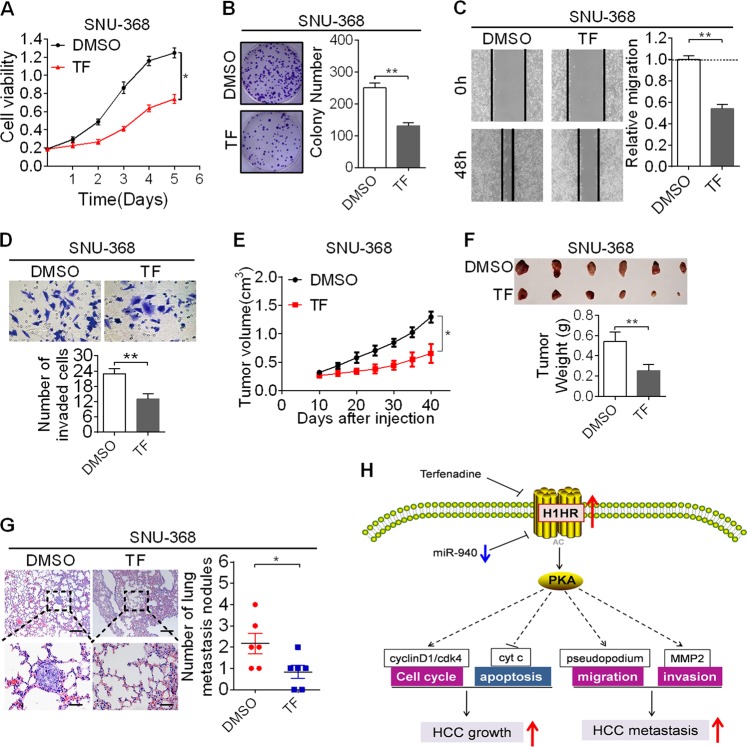
The H1HR inhibitor TF exhibits therapeutic effects on HCC in vitro and in vivo. **a**, **b** MTS and colony formation assays to evaluate the cell growth abilities of SNU-368 cells treated with 4 µM TF or dimethyl sulfoxide (DMSO) as indicated. **c**, **d** Wound healing and transwell matrigel invasion assays to evaluate the migration and invasion abilities of HLE cells treated with 4 µM TF or DMSO as indicated. **e** Subcutaneous tumor growth curve of HCC cells in nude mice treated with TF (20 µg/mice) or DMSO by intratumor injection. **f** Dissected tumors from sacrificed mice are shown in upper panel. Weight of the subcutaneous xenograft tumor is shown in lower panel. **g** Pulmonary metastasis is demonstrated by hematoxylin and eosin staining (scale bar, 50 μm). **h** Schematic diagram depicting the effects of H1HR on the growth and metastasis of HCC cells and their underlying mechanisms. *P < 0.05; **P < 0.01

To explore the therapeutic role of H1HR inhibition on HCC, we first investigated the effects of terfenadine (TF) on HCC cell growth and found that treatment with TF significantly inhibited cell proliferation and colony formation in SNU-368 cells (Fig. 8a, b). In addition, TF treatment also significantly decreased the migration and invasion abilities of SNU-368 cells (Fig. 8c, d). Then, we tested the in vivo effects of TF treatment on tumor growth. TF injection significantly inhibited the growth of xenograft tumors developed from SNU-368 cells (Fig. 8e, f) and significantly inhibited the lung metastasis of SNU-368 cells (Fig. 8g). These results indicate that inhibition of H1HR by a specific antagonist may be a promising novel therapeutic strategy for HCC. On the contrary, treatment with histamine-trifluoromethyl-toluidide derivative (HTMT) (H1HR agonist) significantly increased the growth and metastasis abilities of HLE cells both in vitro and in vivo (Supplementary Fig. S4A–G).

To further explore the potential effect of histamine in H1HR-regulated HCC growth and metastasis, SNU-368 and HLE cells with different levels of H1HR were treated with histamine for 24 h at a concentration of 10 μM. As shown in the Supplementary Fig. S5A, B, histamine treatment significantly increased the growth of HLE and SNU-368 cells, especially when H1HR was overexpressed in HLE cells, whereas slightly increased the growth of SNU-368 when H1HR was knockeddown. Similar results were also obtained when scratch wound healing and transwell matrigel invasion assays were applied (Supplementary Fig. S5C, D), showing that histamine treatment significantly increased both the migration and invasion of HLE and SNU-368 cells, especially when H1HR was overexpressed in HLE cells, whereas slightly increased the migration and invasion of SNU-368 when H1HR was knocked down. These results collectively indicate that histamine promotes the growth and metastasis of HCC cells by acting on H1 plasma membrane receptor, while H1HR could also play a histamine-independent role in the promotion of HCC growth and metastasis.

## Discussion

Recently, high expression levels of histamine receptors have been reported in different human cancers including colon cancer, melanoma, and breast cancer [22]. In the present study, we found that H1HR was frequently upregulated in HCC and contributed to tumor progression. Our results suggest that H1HR overexpression may serve as a valuable new prognostic marker for HCC patients. A previous search for prognostic value of H1HR in the PrognoScan database (a database for meta-analysis of the prognostic value of genes) showed that H1HR is associated with the prognosis of many types of cancers including hematological and solid cancers. In addition, we found that H1HR was expressed not only in HCC cells but also in infiltration myeloid cells. TCGA based analysis also revealed a significantly positive correlation between the expression of H1HR and CD68, indicating that H1HR overexpression may facilitate the infiltration of myeloid cells into HCC tissue. Furthermore, Our results from TCGA-based survival analysis indicated that overexpressed H1HR predicted a poor survival only in HCC patients with high CD68 expression levels, but not in HCC patients with low CD68 expression levels, which further suggested that increased infiltration of myeloid may aggravate the oncogenic effects exerted by H1HR in HCC. However, the relationship between the expression of H1HR and prognosis varied in different cancers, even in the same cancer from different databases [23]. This may be explained by the fact that the function of H1HR in different tumor types may be multidimensional, and it may not simply function as just an oncogene or tumor suppressor.

Histamine is a neurotransmitter that is involved in the regulation of a variety of physiological processes including cell proliferation, differentiation, and immune responses [24]. Consistently, previous studies in breast cancer [25, 26] and cholangiocarcinoma [27] also reported the increased levels of histamine in tumor tissues, which promoted tumor growth and angiogenesis. Unlike histamine, the functional roles of histamine receptors are tumor type-specific. In some types of cancer, histamine receptors promote tumor growth and metastasis. For example, several studies have indicated that H2HR, H3HR, and H4HR exert proproliferative effects on human melanoma, glioblastoma, and colon cancer cells, respectively [28–30]. In contrast, Meng et al. reported an antiproliferative effect of H4HR. [31] in human cholangiocarcinoma. Our present study demonstrate that H1HR overexpression promotes HCC growth by inducing cell cycle progression and reducing cell apoptosis, which is consistent with a previous study in human melanoma cells, showing that H1HR contributes to the reduced apoptosis of human melanoma cells [32]. Similarly, histamine receptors also play dual roles in tumor metastasis. For example, Lin et al. observed a metastasis-promoting role for H3HR in glioblastoma [29]. In contrast, Meng et al. [31] and Cai et al. [33] showed that activation of H4HR suppressed the metastasis of human cholangiocarcinoma and nonsmall cell lung cancer. In the present study, we showed that H1HR promoted the metastasis of HCC cells both in vitro and in vivo. EMT is a key process in metastasis formation during malignant progression. Previous studies have shown that H3HR and H4HR affect tumor metastasis by regulating the EMT [29, 33, 34], whereas data from our study showed that H1HR promoted migration and invasion of HCC by inducing lamellipodia formation and up-regulating MMP-2, but not by regulating the EMT. Possible explanations for these differences include different histamine receptor and tumor origins. Remarkably, we found that histamine treatment significantly increased both the growth and metastasis of HCC cells, especially when H1HR was overexpressed, whereas slightly increased when H1HR was knocked down. These results collectively indicate that histamine promotes the growth and metastasis of HCC cells by acting on H1 plasma membrane receptor, while H1HR could also play a histamine-independent role in the promotion of HCC growth and metastasis.

It has been demonstrated that H1HR stimulates phospholipase to generate IP and DAG, leading to activation of PKC [16, 17]. In addition, H1 receptor activation also stimulates AC to generate cAMP, which in turn activates PKA [20, 35]. Mechanistically, our results demonstrated that the activation of PKA but not PKC was involved in H1HR-mediated HCC growth and metastasis. However, considering that other signaling pathways, such as the mitogen-activated protein kinases and protein kinase B, can also be activated by H1HR [36], we cannot rule out the possibility that these pathways may be involved in this process; this theory needs further clarification.

MiR-940 is a frequently downregulated tumor suppressor in several types of cancer, including HCC. For example, Xu T et al. have demonstrated in glioma that miR-940 was downregulated in clinical samples and glioma cell lines, and suppressed tumor metastasis [37]. Rajendiran et al. have reported that miR-940 is downregulated significantly in prostate cancer and suppresses cell migration and invasion [38]. In HCC, miR-940 was also found to be significantly decreased in the HCC tissues and cell lines, and function as a tumor suppressor through inhibition of invasion and migration of HCC cells [39, 40]. Consistently, our present study showed that miR-940 was involved in the overexpression of H1HR in HCC cells. These results collectively suggest that miR-940 functions as an important tumor suppressor in HCC. However, we still cannot rule out the possibility that other factors may also contribute to the elevated expression of H1HR in HCC, such as genetic and epigenetic alterations, which still needs further confirmation.

TF is an antihistamine reagent (targeting the H1 receptor) that is widely used for the treatment of allergic conditions. Several previous studies have indicated that people with allergy showed an increased risk of certain types of cancer [41, 42], although in several other types of cancer, allergies may have the opposite effect and help protect against cancer [43, 44]. These findings suggest that the association between allergies and cancer risk is complex, the risk of developing cancer could depend on the specific malignancy, and H1HR may be a feasible target in cancer prevention. However, the potential use of H1HR antagonists to treat liver cancer has been unexplored until now. Our data clearly showed that TF treatment significantly inhibited the growth and metastasis of HCC both in vitro and in vivo, indicating that inhibition of H1HR by TF may be a promising novel therapeutic strategy for treating HCC.

In summary, the results of this study indicate that upregulated H1HR promotes both the growth and metastasis of HCC by inducing cell cycle progression, lamellipodia formation, and MMP-2 production and by suppressing cell apoptosis. These findings suggest that H1HR plays an important oncogenic role in HCC and may be a potential therapeutic target for HCC treatment in the future.

## Materials and methods

### Reagents

The PKA inhibitor H89 and activator 8-bromo-cAMP were purchased from Beyotime Biotechnology (Cat. #S1643; Beijing, China) and Sigma-Aldrich (Cat. B-5380; St. Louis, MO), respectively. The H1HR inhibitor TF and H1HR agonist was purchased from Abcam (Cambridge, UK). Histamine was purchased from Sigma-Aldrich. The PKC kinase activity kit (ab139437) and PKA kinase activity kit (ab139435) were purchased from Abcam (Cambridge, UK). The primary antibodies used in this study and their working concentrations were listed in Supplementary Table 3. The sequences of primers and siRNAs used in this study were listed in Supplementary Table 4.

### Cell viability assay

Cell viability was examined by the MTS assay (G3581; Promega Corporation, Madison, WI) according to the manufacturer’s instructions. The microplates were read in a spectrophotometer at a wavelength of 490 nm.

### Colony formation assay

The transiently transfected HCC cells (SNU-368 and HLE) were collected and seeded (1 × 10^3^/well) in six-well plates and cultured for 2 weeks. Colonies were counted after fixing in 70% ethanol staining with 5% crystal violet.

### Cell cycle analysis

The transiently transfected HCC cells (SNU-368 and HLE) were fixed in 70% ethanol for 24 h, and then labeled with 50 μg/ml of propidium iodide (PI; BestBio, shanghai, China). Cell cycle profiles were analyzed on a flow cytometry (Beckman, Fullerton, CA).

### Apoptosis assays

Cell apoptosis was analyzed by flow cytometry and terminal deoxynucleotidyl transferase-mediated dUTP nick-end labeling (TUNEL) assay as previously described [45]. Nuclei with clear green staining were considered TUNEL-positive apoptotic cells. The apoptosis index was calculated as the percentage of TUNEL-positive nuclei after counting at least 500 cells.

### Wound healing assay

Cell migration was assessed using a wound healing assay. Briefly, when the cells reached 80% confluency in six-well plates, three scratch wounds in each well were made using plastic pipette tips (100 μL pipette tips; Eppendorf). The wound closure was observed after 48 h and photographs were taken at 0 and 48 h.

### PKC and PKA activity assay

The activities of PKC and PKA were examined using a PKC and PKA enzyme activity kit, respectively. Briefly, cells were washed twice in ice-cold phosphate-buffered saline and lysed on ice for 10 min. Then, the lysates were centrifuged at 13,000 rpm for 15 min and the clear supernatant extract was analyzed for kinase activity according to the manufacturer’s protocol. Finally, the microplates were read in a spectrophotometer at a wavelength of 450 nm.

### Statistical analysis

Experiments were repeated three times when appropriate. Data were represented as the mean ± standard error of the mean. SPSS 17.0 software (SPSS, Chicago, IL) was used for all statistical analyses and *P* values < 0.05 were considered statistically significant. Paired or unpaired *t* tests were used for comparisons between two groups where appropriate. Correlations between measured variables were tested by Pearson or Spearman rank correlation analyses. OS and RFS in relation to expression were evaluated by the Kaplan–Meier survival curve and the log-rank test.

## Supplementary information

SUPPLEMENTAL MATERIAL
