# Supplementary material for: Upregulation of histamine receptor H1 promotes tumor progression and contributes to poor prognosis in hepatocellular carcinoma
Source: Oncogene. 2019 Nov 18;39(8):1724–38. doi: 10.1038/s41388-019-1093-y (PMC7033043; doi:10.1038/s41388-019-1093-y)
Supplement: Supplementary file 1 — SUPPLEMENTAL MATERIAL [file 41388_2019_1093_MOESM1_ESM.docx]

**Supplemental information**

**Upregulation of histamine receptor H1 promotes tumor progression and contributes to poor prognosis in hepatocellular carcinoma**

**Supplemental materials and methods**

**Cell culture and tissue collection**

The human HCC cell lines SNU-368 and HLE were purchased from the American Type Culture Collection (Manassas, VA) and routinely cultured in RPMI-1640 medium (Gibco, Gaithersburg, MD) or Dulbecco’s Modified Eagle Medium (DMEM; Gibco) supplemented with 10% fetal bovine serum (FBS; HyClone Laboratories, Inc., Logan, UT) at 37°C in a humidified incubator. In addition, 217 paired human HCC tumor and peritumor tissue samples were obtained as previously described[[1](#_ENREF_1), [2](#_ENREF_2)]. This study was approved by the Ethics Committee of the Fourth Military Medical University (Xi’an, China), and written consents were obtained from all patients involved.

**Western blotting and immunohistochemistry**

Total protein in HCC tissues and cell lines was separated by sodium dodecyl sulfate-polyacrylamide gel electrophoresis (SDS-PAGE). The proteins in SDS-PAGE were transferred onto polyvinylidene difluoride membranes. The membranes were incubated with specific primary antibodies in the blocking solution overnight, and then incubated with horseradish peroxidase-conjugated secondary antibody at room temperature for 2 h. Proteins of interest were detected using an enhanced chemiluminescence system. Antibodies and their dilutions are listed in **Supplementary Table 3**. The fold change between tumor and adjacent nontumor tissues was log2-transformed for further analysis.

For immunohistochemistry (IHC), tissues were fixed in 10% of formalin and embedded in paraffin. Immunohistochemistry was performed on four-micrometer paraffin sections using a ready-to-use IHC kit (#859043; Invitrogen). The expression level of target proteins were independently evaluated by two pathologists according to the proportion and intensity of positive cells within five microscopic visual fields per slide as previously described[[3](#_ENREF_3)].

**Overexpression and knockdown of target genes**

To overexpress H1HR, the DNA sequence encoding human H1HR was PCR-amplified from cDNA of HLE cells and then cloned into the pcDNA™3.1 expression vector. For transient knockdown of H1HR, two small interfering RNAs (siRNAs) against the H1HR gene were designed and transfected with Lipofectamine 2000 (11668019; Invitrogen, Carlsbad, CA). Empty vector and scrambled siRNA were used as controls in the H1HR overexpression and transient knockdown experiments, respectively. For generation of short hairpin RNA (shRNA) expression vectors, an shRNA containing specific sequences targeting the human H1HR mRNA sequence (5'-GGATCAGATGTTAGGTGAT-3') was cloned into the pSilencer™ 3.1-H1 puro vector (Ambion). A control shRNA (5'-UUCUCCGAACGUGUCACGUTT-3') was also cloned into the pSilencer™ 3.1-H1 puro vector, which was used as a silencing negative control.

For transfection, SNU-368 and HLE cells were seeded in 6-well plates to 60-80% confluency. Then the vectors were transfected into HCC cells using Lipofectamine 2000 according to the manufacturer’s protocol. Stable transfectants were selected in G418 sulfate (A1720, Sigma-Aldrich) for 4 weeks. The siRNAs sequences (GenePharma, Shanghai, China) and scrambled siRNA are provided in **Supplementary Table 4**. The miRNAs used in this study were purchased from GenePharma. Lipofectamine (Invitrogen, Shanghai, China) was used for transfection according to the manufacturer’ s instructions.

**Quantitative real-time PCR**

Total RNA was extracted from HCC tissue samples or cultured cells using the TRIzol Reagent (Invitrogen), and reverse transcription was performed using the PrimeScript RT Reagent kit with gDNA Eraser (RR047A; Takara Bio, Kusatsu, Japan) according to the manufacturer’s instructions. Quantitative real-time PCR (qRT-PCR) was performed using SYBR 2×qPCR Master Mix (Everbright USA Inc., Redmond, WA). The cycling parameters were 95°C for 15 s, 55°C for 15 s and 72°C for 15 s for 40 cycles. A melting-curve analysis was performed to check the specificity of PCR. The relative expression levels of target genes were determined using the 2^−△△CT^ method. GAPDH served as an internal control. The expression level was normalized to the fold change detected in the corresponding control cells, which was defined as 1.0. For the mRNA expression level of target genes in HCC tissue samples, the fold change between tumor and adjacent nontumor tissues was log2-transformed for further analysis. Primer sequences are listed in **Supplementary Table 4**.

**Transwell migration and matrigel invasion assays**

For the migration assay, the transiently transfected HCC cells (SNU-368 and HLE) were seeded into the upper chamber of a transwell insert (Corning Falcon; Corning, NY) and then placed into the transwell containing DMEM with 10% FBS in the lower chamber. For the invasion assay, cells were seeded in the upper chamber that was pre-coated with 10 mg/mL growth factor-reduced Matrigel (BD Biosciences; Franklin Lakes, NJ). After incubation for 48 h, cells that migrated onto the lower surface of the filter were fixed and stained with crystal violet. Migration and invasion were determined by counting cells in five microscopic fields per well.

**In vivo assays for tumor growth and metastasis analysis**

Male athymic 5-week-old Balb/c nude mice with an average body weight of 18-22 g were housed under standard conditions and randomly divided into groups (6 mice/group). For the tumor growth assay, 1×10^7^ SNU-368-shH1HR and SNU-368-shCtrl cells or HLE-EV and HLE-H1HR cells were injected subcutaneously into dorsal right flank of the Balb/c nude mice (6 mice/group). Tumor volume (mm3) was assessed every 5 days in a 40-day period. The mice were sacrificed in the fourth week, after which the tumor nodules were harvested and photographed. For in vivo metastasis assay, 2.0×10^6^ cells were injected into the tail vein of nude mice. After 6 weeks, the mice were sacriﬁced and lungs were dissected and prepared for standard histological examination. All animal experimental procedures were approved by the Ethics Committee of the Fourth Military Medical University.

**Supplemental figures**

**Figure S1. Increased infiltration of myeloid may aggravate the oncogenic effects exerted by H1HR in HCC.**(**A**) Multiple staining of H1HR, GPC3 and CD68 in HCC tissues by IHC analysis (scale bar, 50 μm). (**B**) Correlation analysis between the expression levels of H1HR and CD68 in HCC tumor tissues based on the data from TCGA. (**C and D**) TCGA-based survival analysis in HCC patients with different levels of CD68 and H1HR.

**
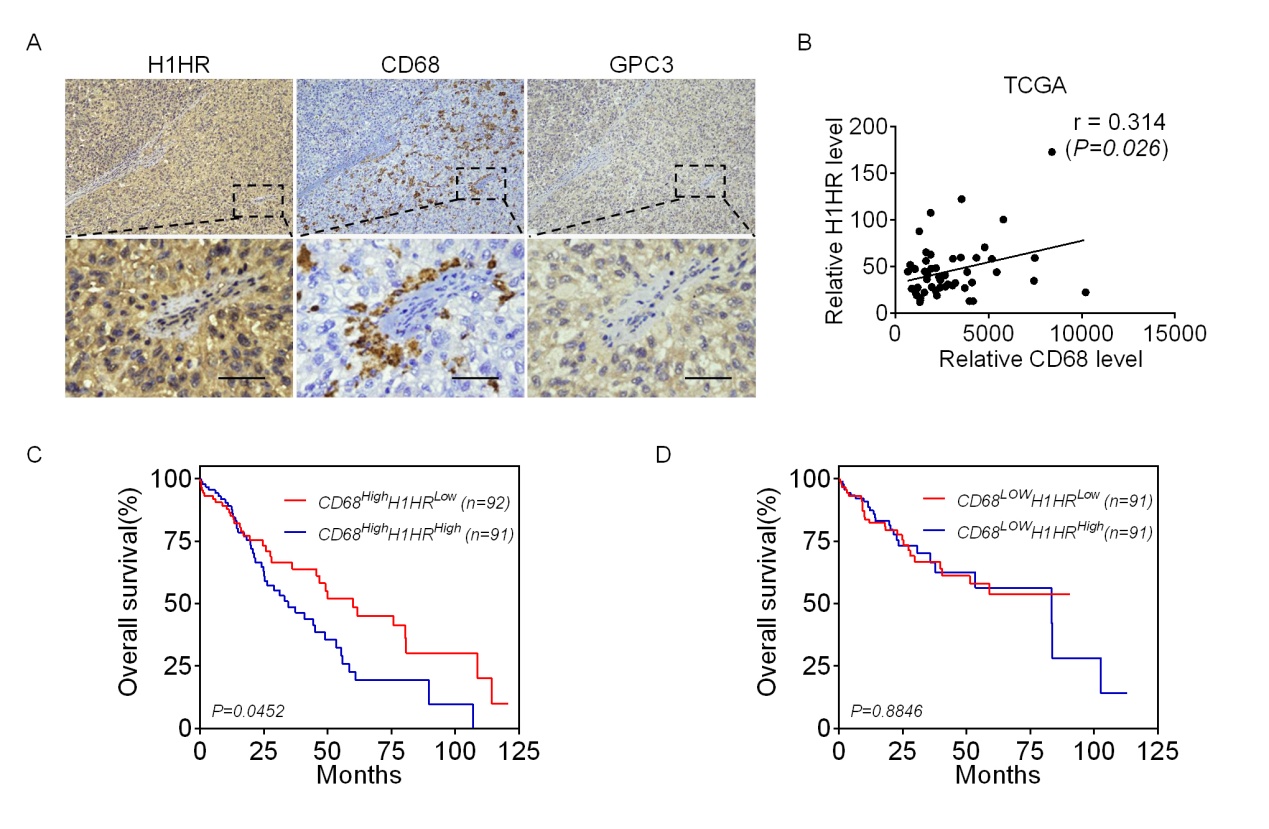
**

**Figure S2. H1HR expression in different HCC cell lines treated as indicated**. **(A and B)** qRT-PCR (**A**) and western blot (B and C) analyses of H1HR expression levels in seven HCC cell lines. (**C and D**) qRT–PCR and western blot analyses for H1HR expression were performed in SNU-368 and HLE cells treated as indicated (siH1HR-1 and siH1HR-2 siRNAs against H1HR; siCtrl, control siRNA; H1HR, expression vector encoding H1HR; EV, empty vector). (**E and F**) qRT–PCR and western blot analyses for H1HR expression in SNU-368 and HLE cells treated as indicated (shH1HR, shRNA against H1HR; shCtrl, control shRNA; H1HR, expression vector encoding H1HR; EV, empty vector).


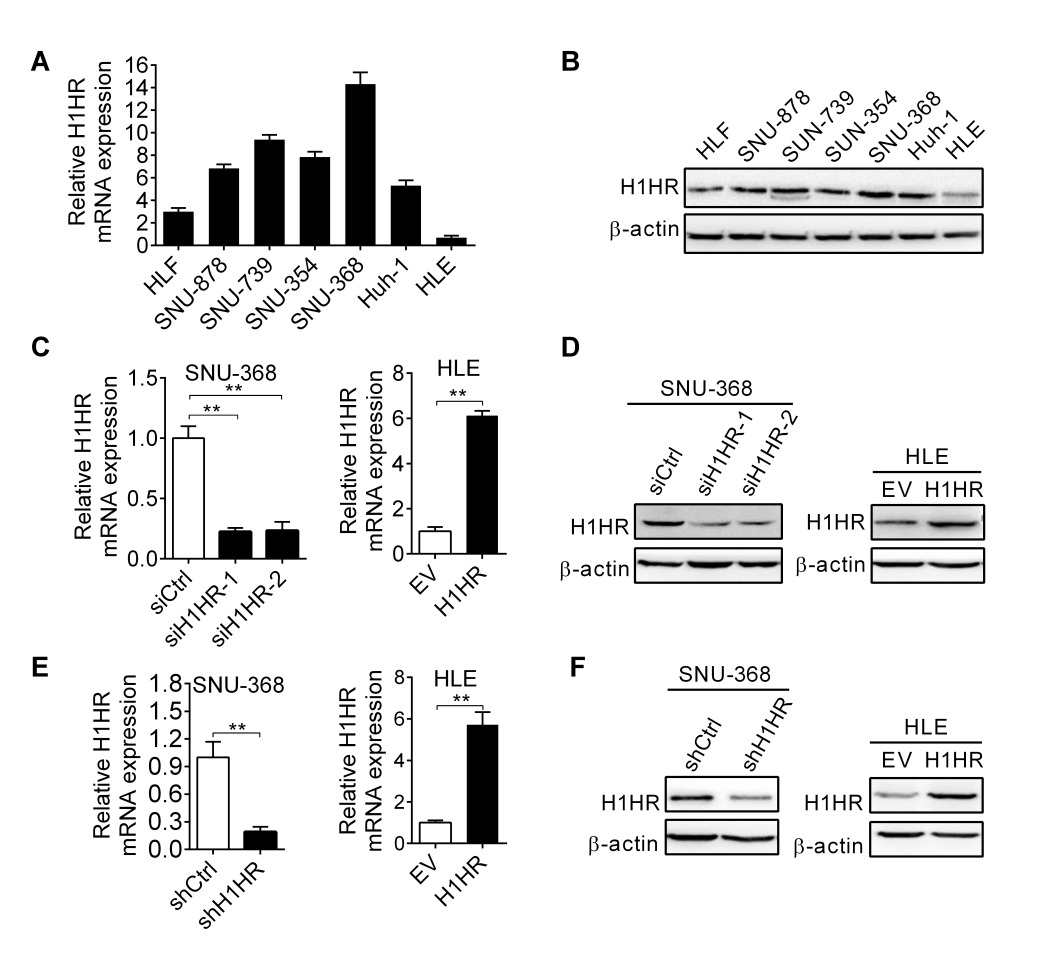


**Figure S3. Western blot analysis for the expressions of H1HR and MMP2 in HLE cells treated as indicated**.

**
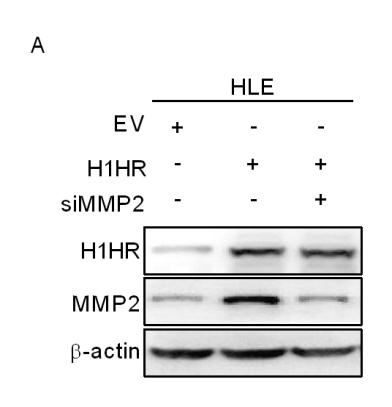
**

**Figure S4. HTMT promotes HCC growth and metastasis of HCC both *in vitro* and *in vivo*.** (A and B) MTS and colony formation assays to evaluate the cell growth abilities of HLE cells treated with 10 µM HTMT or dimethyl sulfoxide (DMSO) as indicated. (C and D) Wound healing and transwell matrigel invasion assays to evaluate the migration and invasion abilities of HLE cells treated with 10µM HTMT or DMSO as indicated. (E) Subcutaneous tumor growth curve of HCC cells in nude mice treated with HTMT (10 µg/mice) or DMSO by intratumor injection. (F) Dissected tumors from sacrificed mice are shown in upper panel. Weight of the subcutaneous xenograft tumor is shown in lower panel. (G) Pulmonary metastasis is demonstrated by hematoxylin and eosin staining (scale bar, 50 μm). *, *P*< 0.05; **, *P*< 0.01.

**
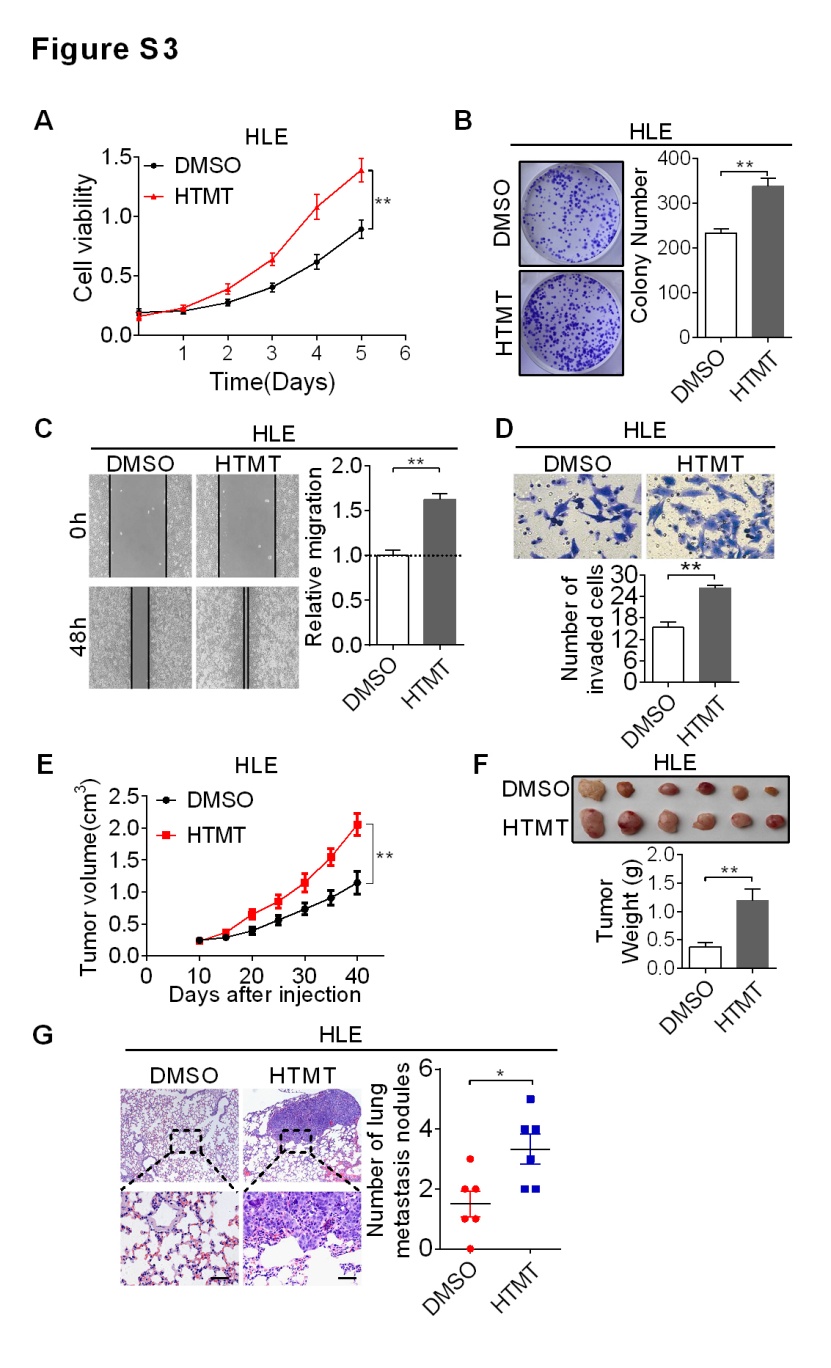
**

**Figure S5. Histamine promotes HCC cell growth, migration and invasion of HCC cells in vitro.** (A) The MTS cell viability assay was performed in SNU-368 and HLE cells treated with histamine for 24 hours at a concentration of 10 μM. (B) Colony formation assay in SNU-368 and HLE cells treated with histamine for 24 hour at a concentration of 10 μM. (C) Scratch wound healing assay for cell migration abilities in SNU-368 and HLE cells with the indicated treatments. (D) Transwell matrigel invasion assay for cell invasion ability of SNU-368 and HLE cells with the indicated treatments. *, P< 0.05; **, P< 0.01.

**
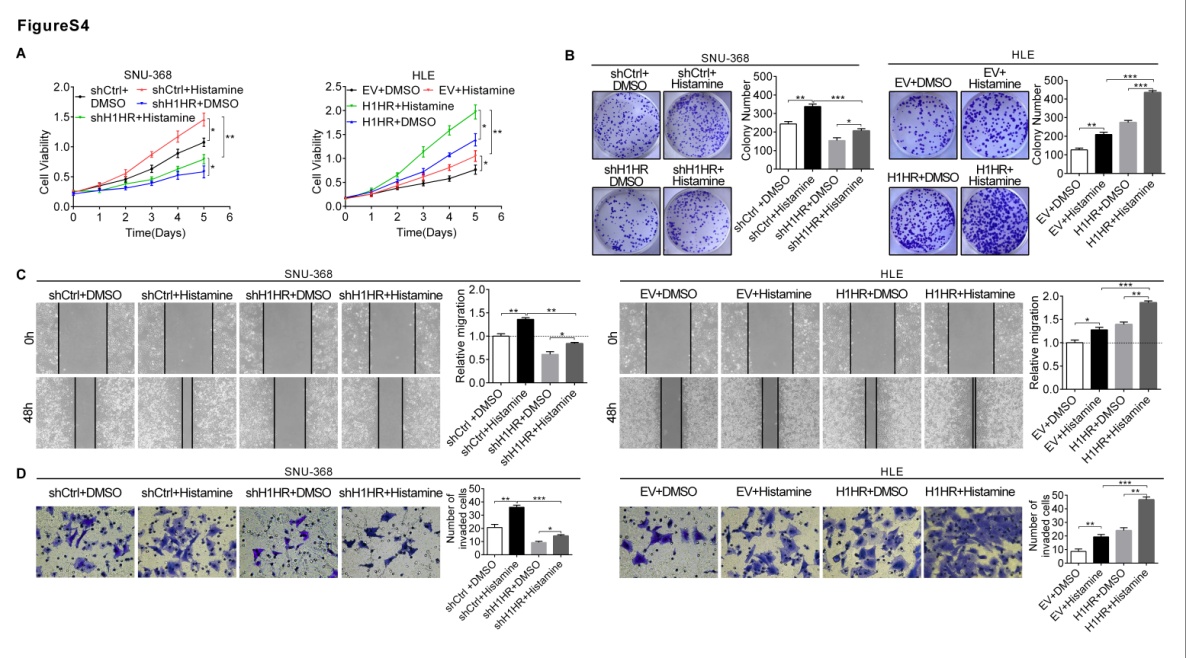
**

**Supplementary Tables**

**Supplementary Table 1. Relationship between H1HR expression and clinicopathologic features of HCC patients.**

| Variables | No. of cases | H1HR expression | | *P* value |
| --- | --- | --- | --- | --- |
|  |  | Low | High |  |
| All | 217 | 108 | 109 |  |
| **Age** |  |  |  | 0.198 |
| <55 | 101 (46.5%) | 55 | 46 |  |
| >=55 | 116 (53.5%) | 53 | 63 |  |
| **Gender** |  |  |  | 0.143 |
| Female | 27 (12.4%) | 17 | 10 |  |
| Male | 190 (87.6%) | 91 | 99 |  |
| **HBsAg** |  |  |  | 0.238 |
| Negative | 19 (8.8%) | 7 | 12 |  |
| Positive | 198 (91.2%) | 101 | 97 |  |
| **AFP (ug/ml)** |  |  |  | 0.152 |
| <200 | 116 (53.5%) | 65 | 51 |  |
| >=200 | 101 (46.5%) | 43 | 58 |  |
| **Maximum diameter of** lesion |  |  |  | <0.001 |
| <5 | 181 (83.4%) | 96 | 85 |  |
| >=5 | 36 (16.6%) | 12 | 24 |  |
| **PVTT** |  |  |  | 0.242 |
| No | 196 (90.3%) | 95 | 101 |  |
| Yes | 21 (9.7%) | 13 | 8 |  |
| **TNM stage** |  |  |  | 0.031 |
| I+ II | 176 (81.1%) | 103 | 73 |  |
| III+ IV | 41 (18.9%) | 5 | 36 |  |
| **Differentiation grade** |  |  |  | 0.265 |
| I+ II | 70 (32.3%) | 31 | 39 |  |
| III | 147 (67.7%) | 77 | 70 |  |
| **Treatment** |  |  |  | 0.054 |
| Hepatectomy | 163 (75.1%) | 75 | 88 |  |
| Hepatectomy+ adjuvant TACE | 54 (24.9%) | 33 | 21 |  |

**Abbreviations**: HCC, hepatocellular carinoma; HBsAg, hepatitis B surface antigen; AFP, α -fetoprotein; PVTT, portal vein tumor thrombosis; TNM, tumor-nodes-metastases; TACE, transcatheter arterial chemoembolization; *P value < 0.05 was considered statistically significant.

**Supplementary Table 2. Sequences of the top ten predicted miRNAs targeting H1HR.**

| **Gene Symbol** | **Uniprot** | **microRNA** | **Integrated Score** | **Number of Sources** | **Score Class** |
| --- | --- | --- | --- | --- | --- |
| HRH1 | P35367 | has-miR-24-3p | 0.630956730743425 | 15 | Very High |
| HRH1 | P35367 | has-miR-940 | 0.579693159695994 | 16 | Very High |
| HRH1 | P35367 | has-miR-518a-5p | 0.575195877373939 | 15 | Very High |
| HRH1 | P35367 | has-miR-29c-3p | 0.545979330839404 | 14 | Very High |
| HRH1 | P35367 | has-miR-129-5p | 0.530972589298945 | 15 | Very High |
| HRH1 | P35367 | has-miR-527 | 0.519378122233337 | 14 | Very High |
| HRH1 | P35367 | has-miR-577 | 0.517919355566081 | 13 | Very High |
| HRH1 | P35367 | has-miR-29b-3p | 0.511042580090234 | 14 | Very High |
| HRH1 | P35367 | has-miR-506-3p | 0.489784923523227 | 11 | Very High |
| HRH1 | P35367 | has-miR-511-3p | 0.489144291959091 | 14 | Very High |

**Supplementary Table 3. Primary antibodies used for Western blotting and IHC analysis.**

| **Antibody** | **Company (Cat. No.)** | **Working Concentration Dilutions** |
| --- | --- | --- |
| H1HR | Abcam(ab154158) | WB: 1/500 IHC:1/100 |
| Caspase-9 | Proteintech(66169-1-Ig) | WB: 1/800 |
| Caspase-3 | Proteintech(25546-1-AP) | WB: 1/800 |
| β-actin | Beijing TDY(TDY051F) | WB: 1/3000 |
| Cyclin D1 | Proteintech(60186-1-AP) | WB: 1/800 |
| CDK4 | Proteintech(11026-1-AP) | WB: 1/500 |
| Cyto C | Proteintech(10993-1-AP) | WB: 1/500 |
| COX IV | ABGENT(#AP9153a) | WB: 1/750 |
| MMP2 | Proteintech(10373-2-AP) | WB: 1/800 IHC:1/200 |
| MMP9 | Proteintech(10375-2-AP) | WB: 1/800 |
| E-cadherin | Proteintech(20874-1-AP) | WB: 1/500 |
| N-cadherin | Cell Signaling(13116) | WB: 1/500 |
| Vimentin | Proteintech(10366-1-AP) | WB: 1/800 |
| ZO-1 | Proteintech(21773-1-AP) | WB: 1/500 |
| p-CREB | Cell Signaling(#9198) | IHC:1/200 |
| Ki67 | MAB(MAB-0129) | IHC:1/200 |

**Supplementary Table 4. Sequences of primers and siRNAs.**

| **1. Primers used in q-PCR analysis** | | | | | |
| --- | --- | --- | --- | --- | --- |
| **Gene** | | **Forward Primer** | | **Reverse Primer** | |
| H1HR | | AAGTCACCATCCCAAACCCCCAAG | | TCAGGCCCTGCTCATCTGTCTTGA | |
| MMP1 | | CACAGCTTTCCTCCACTGCTGCT | | GGCATGGTCCACATCTGCTCTTG | |
| MMP2 | | ACCTGGATGCCGTCGTGGAC | | TGTGGCAGCACCAGGGCA | |
| MMP7 | | AAACTCCCGCGTCATAGAAAT | | CCCTAGACTGCTACCATCCG | |
| MMP9 | | TGACAGCGACAAGAAGTG | | CAGTGAAGCGGTACATAGG | |
| E-cadherin | | GCCCCATCAGGCCTCCGTTT | | ACCTTGCCTTCTTTGTCTTTGTTGGA | |
| N-cadherin | | TGGACCATCACTCGGCTTA | | ACACTGGCAAACCTTCACG | |
| Vimentin | | CCTGAACCTGAGGGAAACTAA | | GCAGAAAGGCACTTGAAAGC | |
| ZO-1 | | CACGCAGTTACGAGCAAG | | TGAAGGTATCAGCGGAGG | |
| GAPDH | | GGAGCGAGATCCCTCCAAAAT | | GGCTGTTGTCATACTTCTCATGG | |
| **2. Primers used in gene cloning** | | | | | |
| **Gene** | | **Forward Primer** | | | **Reverse Primer** |
| H1HR | | GGATCAGATGTTAGGTGAT | | | ATCACCTAACATCTGATCC |
| **3. siRNAs** | | | | | |
| **Gene** | **Forward** | | **Reverse** | | |
| siH1HR-1 | GGGACUAUGUAGCCGUCAATT | | UUGACGGCUACAUAGUCCCTT | | |
| siH1HR-2 | GGAUCAGAUGUUAGGUGAUTT | | AUCACCUAACAUCUGAUCCTT | | |
| siMMP2 | UGGAAUAGCUUCUGGAAUUDTdT | | AAUUCCAGAAGCUAUUCCADAdT | | |
| siControl | UUCUCCGAACGUGUCACGUTT | | ACGUGACACGUUCGGAGAATT | | |

**Reference**

1 Li J, Huang Q, Long X, Guo X, Sun X, Jin X *et al*. Mitochondrial elongation-mediated glucose metabolism reprogramming is essential for tumour cell survival during energy stress. *Oncogene* 2017; 36: 4901-4912.

2 Sun XC, Cao HY, Zhan L, Yin C, Wang G, Liang P *et al*. Mitochondrial fission promotes cell migration by Ca2+/CaMKII/ERK/FAK pathway in hepatocellular carcinoma. *Liver Int* 2018; 38: 1263-1272.

3 Huang Q, Zhan L, Cao H, Li J, Lyu Y, Guo X *et al*. Increased mitochondrial fission promotes autophagy and hepatocellular carcinoma cell survival through the ROS-modulated coordinated regulation of the NFKB and TP53 pathways. *Autophagy* 2016; 12: 999-1014.
